# Mesenchymal stromal cells loaded with paclitaxel induce cytotoxic damage in glioblastoma brain xenografts

**DOI:** 10.1186/s13287-015-0185-z

**Published:** 2015-10-06

**Authors:** Simone Pacioni, Quintino Giorgio D’Alessandris, Stefano Giannetti, Liliana Morgante, Ivana De Pascalis, Valentina Coccè, Arianna Bonomi, Luisa Pascucci, Giulio Alessandri, Augusto Pessina, Maria Laura Falchetti, Roberto Pallini

**Affiliations:** Institute of Neurosurgery, Università Cattolica del Sacro Cuore, Largo Agostino Gemelli 8, 00168 Rome, Italy; CNR-Institute of Cell Biology and Neurobiology (IBCN), via del Fosso di Fiorano 64, 00143 Rome, Italy; Institute of Anatomy, Università Cattolica del Sacro Cuore, Largo Agostino Gemelli 8, 00168 Rome, Italy; Institute of Pathology, Università Cattolica del Sacro Cuore, Largo Agostino Gemelli 8, 00168 Rome, Italy; Department of Biomedical, Surgical and Dental Sciences, University of Milan, via Pascal 36, 20133 Milan, Italy; Department of Veterinary Medicine, University of Perugia, via San Costanzo 4, 06126 Perugia, Italy; Department of Cerebrovascular Diseases, Fondazione IRCCS Neurological Institute Carlo Besta, via Giovanni Celoria 11, 20133 Milan, Italy

## Abstract

**Introduction:**

The goal of cancer chemotherapy is targeting tumor cells and/or tumor-associated microvessels with the lowest systemic toxicity. Mesenchymal stromal cells (MSCs) are promising vehicles for selective drug delivery due to their peculiar ability to home to pathological tissues. We previously showed that MSCs are able to uptake and subsequently to release the chemotherapeutic compound Paclitaxel (PTX) and to impair the growth of subcutaneous glioblastoma multiforme (GBM) xenografts. Here we used an orthotopic GBM model *1)* to assess whether PTX-loaded MSCs (PTX-MSCs) retain a tropism towards the tumor cells in the brain context, and *2)* to characterize the cytotoxic damage induced by MSCs-driven PTX release in the tumor microenvironment.

**Methods:**

U87MG GBM cells were fluorescently labeled with the mCherry protein and grafted onto the brain of immunosuppressed rats. In adjacent brain regions, we injected green fluorescent protein-expressing murine MSCs, either loaded with PTX or unloaded. After 1 week survival, the xenografted brain was assessed by confocal microscopy for PTX-induced cell damage.

**Results:**

Overall, MSCs showed remarkable tropism towards the tumor. In rats grafted with PTX-MSCs, the nuclei of U87MG cells showed changes that are typically induced by PTX, including multi-spindle mitoses, centrosome number alterations, and nuclear fragmentation. Multi-spindle mitoses resulted in multinucleated cells that were significantly higher in tumors co-grafted with PTX-MSCs than in controls. Nuclear changes did not occur in astrocytes and neurons surrounding the tumor.

**Conclusions:**

MSCs appear particularly suited for anti-neoplastic drug delivery in the brain since PTX-specific damage of GBM cells can be achieved avoiding side effects to the normal tissue.

**Electronic supplementary material:**

The online version of this article (doi:10.1186/s13287-015-0185-z) contains supplementary material, which is available to authorized users.

## Introduction

The key goal of cancer chemotherapy consists of localizing the drug effect selectively to the tumor microenvironment in order to kill as many cancer cells as possible while producing the lowest collateral toxicity. To achieve this, a significant number of approaches have been investigated in the last 20 years, from the use of toxic immunoconjugates for targeting tumor specific antigens to sophisticated use of nanoparticles or manipulated stem cells for selective drug delivery [1–3].

Glioblastoma multiforme (GBM), the most aggressive brain tumor, is associated with invariably unfavorable prognosis in spite of extensive surgical resection, radiotherapy, and concomitant and adjuvant chemotherapy with temozolomide [4]. Unfortunately, the efficacy of systemic therapies is limited by the blood–brain barrier. There is therefore an urgent need for new vehicles that enable local, persistent delivery of chemotherapeutic drugs.

Mesenchymal stem/stromal cells (MSCs) are adult stem cells first described by Friedenstein et al. [5] as adherent fibroblast-shaped cells in the bone marrow, capable of differentiating into bone. More recently, it has been shown that MSCs can be isolated from various tissues, such as adipose tissue, umbilical cord blood, Wharton jelly, and derma. MSCs are defined as plastic adherent cells, expressing a variety of surface markers (e.g., CD44, CD63, CD105, CD146) with the capacity for in vitro differentiation into osteoblasts, adipocytes, and chondrocytes.

MSCs have recently gained great interest as a therapeutic tool due to their unique biological features, including the ability to home to pathological tissues, to differentiate into various cell types, to secrete bioactive molecules stimulating recovery after tissue damage, and to play immunomodulatory roles. Due to these peculiarities, MSCs represent a great opportunity for cancer therapy. Using transgenic procedures, MSCs have been induced to secrete therapeutic cytokines or growth/inhibitory factors with the capacity to kill cancer cells, both in vitro and in vivo [3, 6–8]. However, genetic manipulation of MSCs in the clinical setting implies risks of pro-tumorigenic effects [9].

Paclitaxel (PTX) is a microtubule poison that arrests cells in mitosis. PTX promotes microtubule assembly and stabilization [10–12], thus leading to activation of the mitotic checkpoint that arrests cells in mitosis. Low concentrations of PTX suppress the rate at which microtubules grow and shrink, without substantially increasing the microtubule polymer mass, while arresting cells in mitosis on multipolar spindles [13]. Cells arrested in mitosis can either die or undergo a process known as mitotic slippage, in which they enter the G1 phase without undergoing anaphase or cytokinesis to produce a single, tetraploid cell. Repeated mitoses in the absence of cytokinesis result in aberrant multinucleated cells eventually undergoing apoptotic death [14, 15].

In a previous work we demonstrated that MSCs without any genetic manipulation are able to uptake and subsequently to release PTX in an amount sufficient to inhibit both tumor and endothelial cell proliferation in vitro and, most importantly, to impair tumor growth in a subcutaneous GBM xenograft model [16]. In brain xenografts, Menon et al. [17] demonstrated that human MSCs possess significant tropism towards U87MG tumor cells.

In the present study we used an orthotopic GBM model to assess whether PTX-loaded MSCs retain a tropism towards the tumor cells and exert a selective anti-tumor effect in the brain environment. We found that PTX-MSCs efficiently migrated from the injection site to the tumor, showing that PTX does not affect MSC tropism toward the tumor. Notably, nearly all PTX-MSCs either penetrated into or located around the tumor. In U87MG xenografts containing PTX-MSCs, we found PTX-induced cytotoxic damage in the tumor cells, including atypical mitoses, abnormal spindles, and abnormal centrosomes, which resulted in chromosome missegregation and aberrant multinucleated cells.

## Materials and methods

### Cell cultures

All cells were cultured at 37 °C in a humidified atmosphere containing 5 % carbon dioxide. The SR4987 murine stromal cell line was established from a long-term bone marrow-derived cell culture of BDF/1 mice (CRL-2028; ATCC). This cell line shows a high degree of stemness [18], expresses vimentin, CD44, CD73, CD105, CD106, Sca1, and CD34, contains 50 % of CD45^+^ cells, and is capable of differentiating into osteocytes and chondrocytes [18, 19]. Cells were grown in Iscove’s modified Dulbecco’s medium (IMDM; Sigma, St. Louis, MO, USA) supplemented with 5 % fetal bovine serum (Gibco by Life Technologies, Waltham, MA, USA) under standard conditions. U87MG GBM cells (HTB14; ATCC) were cultured in Dulbecco’s modified medium (high-glucose Dulbecco’s modified Eagle’s medium (DMEM); Sigma, St.Louis, MO, USA) supplemented with 10 % fetal bovine serum (Gibco) under standard conditions.

### Production of viral stocks and cell infections

U87MG cells expressing the red fluorescent protein mCherry (Cherry-U87MG) and SR4987 MSCs expressing the green fluorescent protein (GFP-MSCs) were obtained by lentiviral transduction as described by Dull et al. [20]. Briefly, viral particles were produced in HEK 293 T cells by transient transfection using Lipofectamine reagent (Life Technologies, Waltham, MA, USA). HEK293T were co-transfected with the lentiviral constructs, pCLLsin.PPT.hPGK.GFP.pre [20] or pLVX-mCherry-C1 (Clonetech Laboratories Inc., Mountain View, CA, USA), together with the packaging plasmids pMDL, pRSV, and VSVG. Supernatants were collected every 24 hours between 48 and 72 hours after transfection, and used in three successive rounds of infection in the presence of 8 μg/ml polybrene. Lentiviral infection occurred with high efficiency, as assessed by red and green fluorescence, so that no enrichment for transduced cells was required.

### Loading of GFP-MSCs with PTX

GFP-MSCs were loaded with 2000 ng/ml PTX (2340 nM; Adipogen, Liestal, Switzerland), as described by Pessina et al. [16]. Briefly, cells were incubated with PTX for 24 hours. At the end of incubation, cells were trypsinized, extensively washed with Hanks Balanced salt solution (HBSS), and seeded in a new flask. After 24 hours, conditioned medium (CM) was collected and used to culture U87MG cells. Parallel cultures of U87MG cells were grown with CM from unloaded MSCs and used as controls. The amount of PTX in the CM was evaluated by high-performance liquid chromatography, as described previously [21].

### MTS assay

The effect of CM from PTX-MSCs on U87MG cell viability was evaluated by the Cell Titer 96 Aqueous One Solution Cell Proliferation Assay (Promega, Madison, WI, USA). U87MG cells were seeded on a 96-well plate (1500 cells/well) and cultured for 5 days in the presence of serial 1:2 dilutions of CM from PTX-MSCs or of PTX (initial concentration = 200 ng/ml). Cell viability was calculated as the ratio between the absorbance of treated and control (sham treated) U87MG cells. Mean and standard deviation values were generated from two biological replicates. Each experiment was performed at least three times. Representative results of a single experiment are shown in Additional file 1. Three independent experiments were consistent.

### U87MG cell treatment with PTX

U87MG cells were seeded on glass slides and cultured in the presence of 20, 50, and 100 nM PTX for 24 hours. U87MG cells were also cultured with CM from SR4987-PTX-MSCs. As negative control, we used CM from unloaded MSCs. After 24 hours, U87MG cells were fixed with 4 % paraformaldehyde for fluorescence microscopy.

### Immunocytochemical analysis

U87MG cells grown on coverslips were washed in 0.1 M phosphate buffer (PB) and fixed with 4 % paraformaldehyde for 20 minutes at room temperature (RT). Fixation was quenched by washing twice. Cells were permeabilized and blocked with PB containing 0.3 % Triton X-100, 5 % normal donkey serum (NDS), and 1 % bovine serum albumin (BSA) for 30 minutes at RT in a humid chamber, and then washed and incubated in humid chamber for 2 hours at RT with primary mouse anti-α-tubulin and rabbit anti-γ-tubulin antibodies (1:250; Sigma-Aldrich, St. Louis, MO, USA) for staining of spindles and centrosomes respectively. Cells were washed and incubated in PB containing 5 % NDS and 1 % BSA with Alexa Fluor 555 donkey anti-mouse and Alexa Fluor 488 donkey anti-rabbit secondary antibodies (1:500; Life Technologies, Waltham, MA, USA) for 1 hour at RT in humid chamber. Cells were washed, incubated with 4′,6-diamidino-2-phenylindole (DAPI, 1:4000; Sigma-Aldrich, St. Louis, MO, USA) for 5 minutes, and washed again before mounting the coverslips with mounting medium. Visualization and acquisition of images were accomplished using a laser confocal microscope (SP5; Leica). Acquired images were analyzed with Leica Application Suite X software.

### Intracranial xenografts of Cherry - U87MG cells and GFP-MSCs

Experiments involving animals were approved by the Ethical Committee of the Catholic University School of Medicine, Rome (Pr. No. FF22). Adult male Wistar rats (200–250 g; Catholic University Breeding Laboratory, Rome, Italy) were used. Beginning 7 days before implantation, the rats were immunosuppressed with subcutaneous injection of cyclosporine (30 mg/kg, three times per week). For cell grafting, the rats were anesthetized with intraperitoneal injection of diazepam (2 mg/100 g) followed by intramuscular injection of ketamine (4 mg/100 g). Animal skulls were immobilized in a stereotactic head frame and three burr holes were made. Two holes were made 1 mm anterior to the bregma 1.5 and 3 mm right of the midline. The third hole was 1 mm posterior to the bregma 2 mm right of the midline. The tip of a 10 μl Hamilton microsyringe was then placed at a depth of 4 mm from the dura and 2 × 10^5^ GFP-PTX-MSCs were injected in each hole anterior to the bregma. In the hole posterior to the bregma, 2 × 10^5^ Cherry-U87MG cells were injected at the same depth from the dura. Control animals were grafted with unloaded GFP-MSCs. An additional group of rats was grafted with 2 × 10^5^ Cherry-U87MG cells alone. After grafting, the animals were kept under pathogen-free conditions in positive-pressure cabinets (Tecniplast Gazzada, Varese, Italy) and observed daily for neurological signs. After 1 week of survival, the rats were deeply anesthetized and transcardially perfused with 0.1 M phosphate-buffered saline (PBS; pH 7.4) followed by 4 % paraformaldehyde in 0.1 M PBS.

### Fluorescence microscopy and immunofluorescence of brain tumor xenografts

The brains were removed, post-fixed in 4 % paraformaldehyde in PB for 3 days, and cryoprotected in PB with 30 % sucrose for 3 days. Coronal sections of the brain (40 μm thick) were blocked in PB with 10 % BSA, 0.3 % Triton X-100 for 45 minutes. Sections were incubated overnight at 4 °C with primary mouse anti-α-tubulin or rabbit anti-γ-tubulin (1:250; Sigma-Aldrich) for staining of spindles and centrosomes respectively, or rabbit anti-GFAP (1:1000; Dako Italia, Milan, Italy), rabbit anti-NeuN (1:400; EMD Millipore, Billerica, MA, USA), or rabbit anti-Iba1 (1:200; Wako Chemicals, Richmond, VA, USA) antibodies, identifying astrocytes, neurons, and microglia respectively. Slices were rinsed and incubated in PB containing 0.3 % Triton X-100 with Alexa Fluor 647 donkey anti-mouse or Alexa Fluor 647 donkey anti-rabbit secondary antibodies (1:500; Life Technologies) for 2 hours at RT. Before mounting, slices were incubated with DAPI (1:4000; Sigma-Aldrich) for 10 minutes. Immunofluorescence was observed with a laser confocal microscope (SP5; Leica) and images were acquired. Image analysis was performed with Leica Application Suite X software.

### Statistical analysis

Results are presented as mean ± standard error of the mean (SEM) and statistically evaluated by one-way analysis of variance (ANOVA) or by Wilcoxon tests. Multiple comparisons were performed using the post-hoc Bonferroni comparison test. The relationship between two variables was assessed using Pearson correlation analysis. All statistical analyses were performed with GraphPad Prism 5 software (GraphPad Software, San Diego, CA, USA).

## Results

### Assessment of cytotoxic effect of PTX

**Fig. 1:**
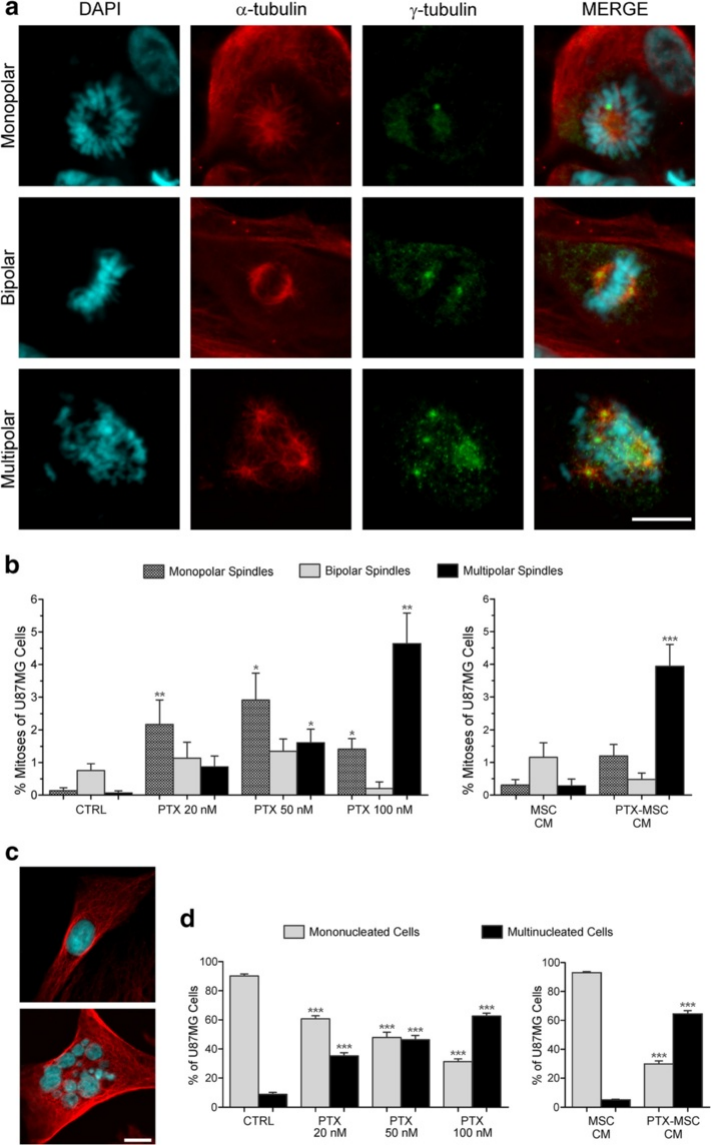
Cytotoxic effect of PTX on cultured U87MG cells. U87MG cells treated with PTX undergo aberrant mitoses with monopolar or multipolar spindles, as assessed by immunostaining of mitotic spindles and centrosomes by anti-α-tubulin and anti-γ-tubulin antibodies, respectively (**a**). The percentage of abnormal spindles is dose dependent. Changes in the percentage of monopolar and multipolar spindle mitoses are significant at 50 and 100 nM PTX (*p <0.05 and **p <0.0005) (**b**) (*left panel*). *n* >1000 cells were counted from each of three independent experiments. Exposure of U87MG cells to the CM of PTX-loaded MSCs induces a strong cytotoxic effect, quantitatively similar to 100 nM PTX, with significant increase of multipolar spindle mitoses (***p <0.0001) (**b**) (*right panel*). As a consequence of multispindle mitoses, the percentage of multinucleated U87MG cells (**c**) (*lower panel*) versus mononucleated U87MG cells (**c**) (*upper panel*) significantly increases (***p <0.0001) both after direct PTX treatment (**d**) (*left panel*) and after exposure to CM from PTX-loaded MSCs (**d**) (*right panel*). *n* >2000 cells were counted from each of three independent experiments. Scale bars = 10 μm. *CM* conditioned medium, *CTRL* control, *DAPI* 4′,6-diamidino-2-phenylindole, *MSC* mesenchymal stem/stromal cell, *PTX* paclitaxel

To assess the cytotoxic effect of PTX, we exposed the U87MG cells for 24 hours to increasing doses of PTX, ranging from 20 to 100 nM. Exposure of U87MG cells to PTX results in a dramatic increase of multispindle mitoses, as assessed by immunohistochemical staining of spindles and centrosomes with anti-α-tubulin and anti-γ-tubulin antibodies respectively (Fig. 1a). The percentage of monopolar and multipolar spindle mitoses increases in a dose-dependent manner (Fig. 1b). While untreated U87MG cells show 0.13 ± 0.09 and 0.06 ± 0.06 % (mean ± SEM) monopolar and multipolar spindle mitoses, respectively, following treatment with 100 nM PTX the percentage of abnormal spindle mitoses in the U87MG cells increases to 1.41 ± 0.34 and 4.63 ± 0.94 % (mean ± SEM) of monopolar and multipolar spindle mitoses, respectively. Of note, the tumor cells exposed to PTX showed a higher number of spindles with respect to control cells (Fig. 1b). For example, the percentage of mitotic figures was significantly higher in U87MG cells treated with 100 nM PTX than in untreated control cells (6.25 ± 1.14 and 0.95 ± 0.27 %; *p* <0.0005, Wilcoxon test). This is probably because PTX stabilizes microtubules preventing their depolymerization, therefore increasing the number of spindled cells, owing to the active mitotic checkpoint which freezes cells in metaphase. As a consequence of multispindled mitoses, the percentage of multinucleated U87MG cells derived from mitotic slippage increased, in a dose-dependent manner, from 8.87 ± 1.35 % (mean ± SEM) of control cells to 62.49 ± 2.06 % (mean ± SEM) of U87MG treated with 100 nM PTX (Fig. 1d; *p* <0.0001, ANOVA test).

Then, we analyzed the cytotoxic effect produced by the CM from PTX-loaded MSCs on U87MG cells. The mouse bone marrow-derived stromal cell line SR4987 has been described to be efficiently loaded with PTX and to be strongly resistant to PTX cytotoxicity [16, 21]. The PTX equivalent concentration, measured by comparing the inhibitory activity of pure PTX and that of CM from SR4987-PTX cells on a sample cell line (MOLT-4), was previously found to be in the range of 33 pg/cell [16]. To determine the effects of CM from PTX-MSCs on U87MG cells, we loaded the SR4987 MSCs with 2000 ng/ml PTX (2340 nM) and added the CM to cultured U87MG cells. The ability of CM from PTX-loaded MSCs to affect U87MG cell viability was addressed by MTS assay, in comparison with U87MG cells treated with increasing PTX doses ranging from 1.56 to 200 ng/ml. Coherently with our previous results [16], CM dilutions of 1:8 caused a reduction of cell viability of up to 80 % with respect to untreated control U87MG cells, with kinetics strictly resembling that of U87MG cells treated with PTX (Additional file 1).

CM from PTX-MSCs caused a striking cytotoxic effect in terms of altered spindle mitoses (Fig. 1b–d). Multispindle mitoses increased from 0.28 ± 0.20 % (mean ± SEM) in U87MG cells cultured with CM from unloaded MSCs to 3.94 ± 0.67 % in U87MG cells cultured with CM from PTX-MSCs (Fig. 1b, right panel; *p* <0.0001, Wilcoxon test). As a consequence of aberrant spindles, multinucleated cells increased from 5.20 ± 0.40 to 64.53 ± 2.25 % (mean ± SEM) (Fig. 1c, lower image and d, right panel; *p* <0.0001, ANOVA test).

It is noteworthy that following treatment with CM from PTX-MSCs, both the percentage of abnormal spindles and the ratio of mononucleated/multinucleated U87MG cells were comparable with those obtained in cells treated with PTX at the higher dose (100 nM) (Fig. 1b, d). U87MG cells exposed to the CM from unloaded MSCs showed an incidence of abnormal spindles and of multinucleated cells which was similar to those seen in control U87MG cells (Fig. 1b, d), indicating that the effect of CM on mitotic events is exclusively due to PTX released in the CM.

### PTX-loaded MSCs home to brain tumor

**Fig. 2:**
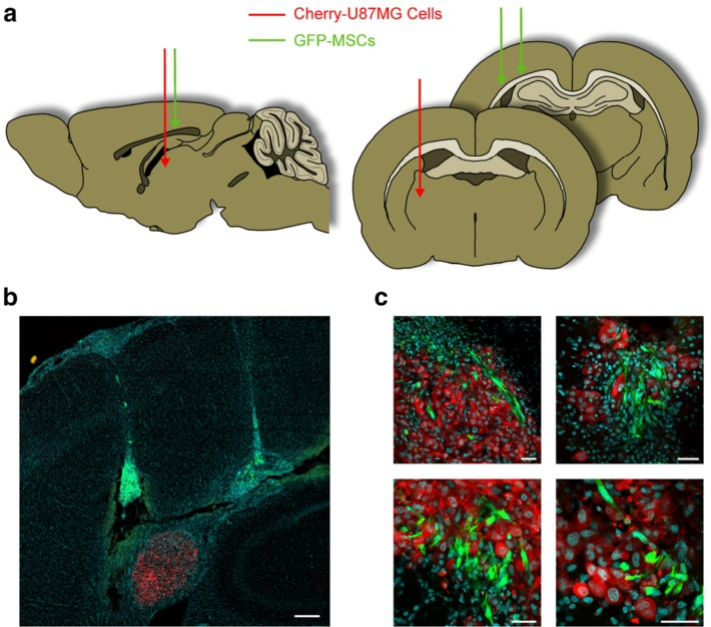
Orthotopic brain tumor xenograft. Cherry-U87MG cells and GFP-MSCs, either unloaded or loaded with PTX, were grafted in the right striatum of immunosuppressed rats. The two cell lines were injected into distinct brain sites (**a**). Low-power picture of a coronal section through the injection sites of GFP-MSCs showing their migration towards the Cherry-U87MG cells. Scale bar = 250 μm (**b**). High-power pictures showing the red tumor that appears to be massively colonized by the green MSCs. Scale bars = 40 μm (**c**). *GFP* green fluorescent protein, *MSC* mesenchymal stem/stromal cell

In a previous study, we demonstrated that MSCs loaded with PTX are able to reduce tumor growth in a subcutaneous U87MG GBM model [16]. To address the ability of PTX-loaded MSCs to migrate towards GBM tumors through the brain environment, we established an orthotopic brain tumor by engrafting red fluorescent Cherry-U87MG cells and green fluorescent GFP-MSCs loaded with PTX onto the right striatum of cyclosporine-immunosuppressed rats (*n* = 6) (Fig. 2a). The two cell populations were engrafted separately in distinct sites that were about 2–3 mm apart. The rationale for this design was to assess whether PTX-loaded MSCs may retain a tropism towards the tumor tissue, which has been described for the unloaded MSCs [22]. Controls included rats inoculated either with Cherry-U87MG plus unloaded MSCs (*n* = 5) or with Cherry-U87MG cells alone (*n* = 6). Fluorescent microscopy analysis of seriated brain sections showed that both PTX-loaded MSCs and unloaded MSCs traveled from the injection site towards the U87MG cells and extensively colonized the tumor xenograft (Fig. 2b, c). There were no differences in the path of brain migration between PTX-loaded and unloaded MSCs. Interestingly, we did not find MSCs in brain areas far from the tumor, thus demonstrating that the PTX-loaded MSCs are able to retain a strong tropism towards the U87MG tumor cells.

### PTX-MSCs exert cytotoxic effect on the tumor cells in vivo

**Fig. 3:**
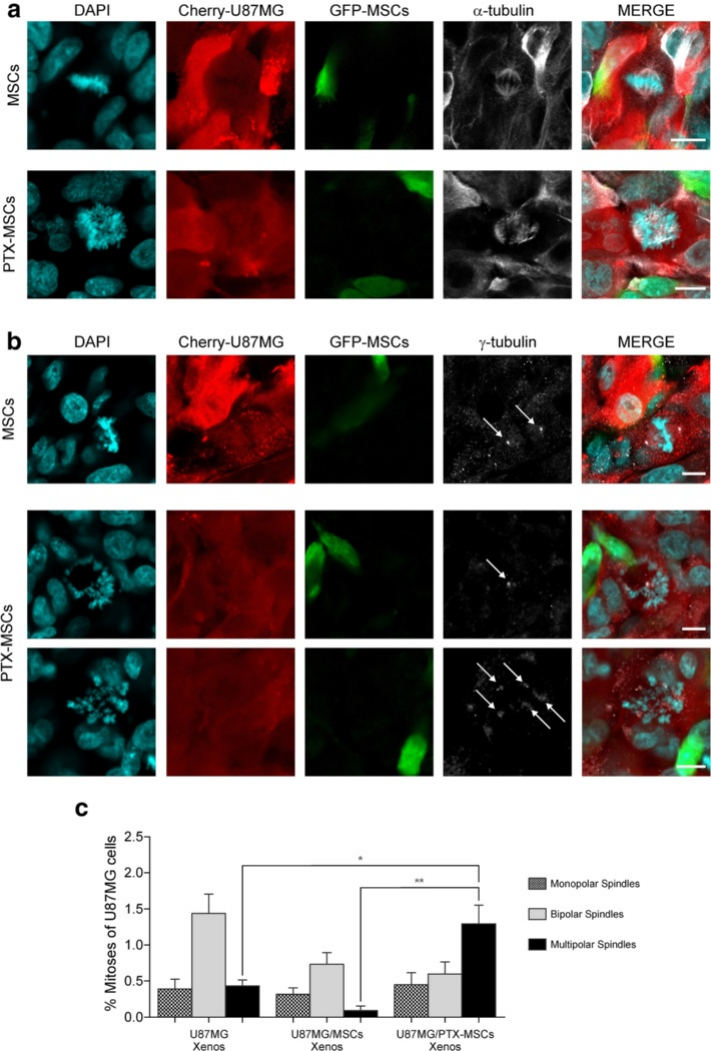
Immunohistochemical characterization of PTX-induced cytotoxic effect in U87MG xenografts. Representative immunostaining with markers of mitotic spindles (α-tubulin) and of centrosome (γ-tubulin) showing mitotic alterations in brain tumor xenografts. In brain xenografts containing PTX-loaded MSCs, there was a strong increase in aberrant spindle configurations, mainly resulting in multipolar spindle mitoses (**a**) and in centrosome number defects (**b**) (*white arrows*). Scale bars = 10 μm. Multipolar spindles of tumor cells in the PTX-MSCs/U87MG grafts are significantly higher with respect both to tumor xenografts derived by injection of U87MG cells alone and to tumors generated by the injection of U87MG cells and unloaded MSCs (**c**). *DAPI* 4′,6-diamidino-2-phenylindole, *GFP* green fluorescent protein, *MSC* mesenchymal stem/stromal cell, *PTX* paclitaxel

**Fig. 4:**
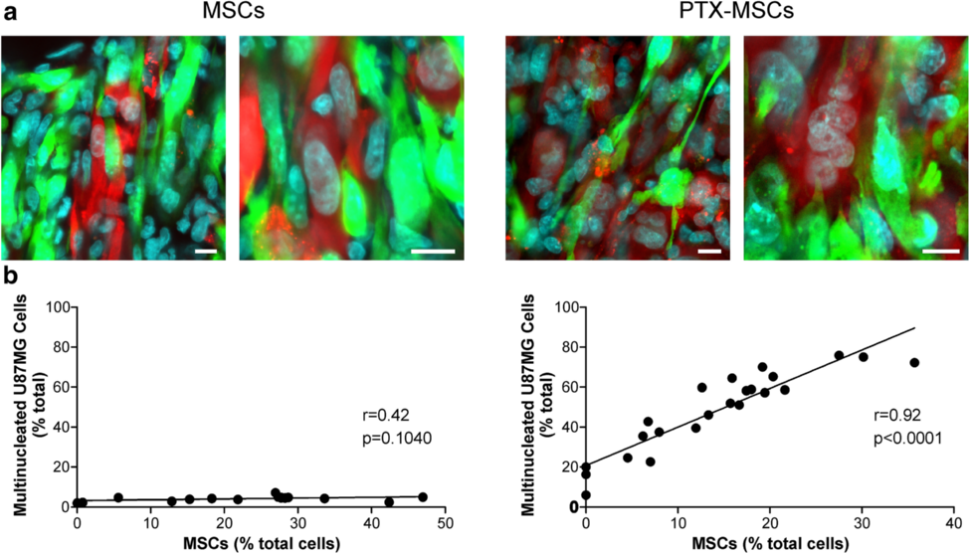
Cytotoxic effect of PTX on U87MG brain xenografts. PTX released into the tumor microenvironment by PTX-loaded MSCs causes cytotoxic effect to the U87MG cells resulting in aberrant, multinucleated tumor cells (scale bars = 10 μm) (**a**). The percentage of multinucleated U87MG cells directly relates to the percentage of PTX-loaded MSCs on the total number of cells *per* field (**b**). *MSC* mesenchymal stem/stromal cell, *PTX* paclitaxel, *r* Pearson correlation coefficient

We then wondered whether PTX-loaded MSCs may induce any cytotoxic effect on xenografted U87MG cells, reproducing a scenario similar to that observed in vitro. Our in vitro data were translated to the in vivo model in order to provide direct evidence that the PTX-loaded MSCs are able to induce a specific cytotoxic effect on the tumor cells. To characterize the cytotoxic damage caused by PTX, we immunostained the mitotic spindles and centrosomes of tumor cells with anti-α-tubulin and anti-γ-tubulin antibodies, respectively. Coherently with the in vitro observations, we found a significant increase of abnormal spindles in the tumor brain xenografts that had been coinjected with PTX-loaded MSCs compared with tumors containing either Cherry-U87MG cells alone (0.43 ± 0.10 %; *p* <0.005, Wilcoxon test) or Cherry-U87MG plus unloaded MSCs (0.09 ± 0.06 %; *p* <0.0005, Wilcoxon test) (Fig. 3c). These results demonstrate that PTX released into the tumor microenvironment by PTX-loaded MSCs increased the amount of abnormal mitoses mostly because of multispindle divisions. We then assessed the mononucleated and multinucleated U87MG cells in xenografts containing either PTX-loaded MSCs or unloaded MSCs (Fig. 4). Multinucleated tumor cells were very rare in brains grafted with U87MG cells alone as well as in the tumors cografted with unloaded MSCs. Conversely, the brain tumors populated by PTX-loaded MSCs showed a remarkable fraction of multinucleated U87MG cells that ranged between 5.97 and 75.86 % (Fig. 4a). In the xenografts containing U87MG cells plus unloaded MSCs, the percentage of multinucleated U87MG cells was very low (3.23 ± 0.32 %, mean ± SEM), and this result was not affected by the density of MSCs that surrounded the tumor cells (Fig. 4b, left panel; Pearson’s correlation coefficient *r* = 0.42). Conversely, the number of multinucleated U87MG cells was significantly related to the density of PTX-MSCs (Fig. 4b, right panel; Pearson’s correlation coefficient *r* = 0.92), demonstrating that the nuclear changes in the tumor cells are a direct consequence of PTX release by MSC-loaded cells. Note that in the U87MG plus PTX-MSCs xenografts, the basal level of multinucleated tumor cells per tissue section was higher than in control U87MG plus unloaded MSC xenografts (Fig. 4b). This result is probably due to PTX-MSCs that lay in adjacent tissue sections and were not counted. We did not observe PTX-induced nuclear changes in the astrocytes as well as in neurons surrounding the brain site where PTX-loaded MSCs had been implanted (Additional file 2).

To assess whether cyclosporine depressed the immune response at similar levels amongst individual rats, we investigated the brain microglia by Iba1 immunofluorescence in the grafted region and, as a control, in the contralateral striatum (Additional file 3) [23]. Compared with the brain region contralateral to the xenograft, where rare resting Iba1-positive cells were sparsely and homogeneously distributed, the grafted striatum showed a remarkable increase of the activated microglial cells. However, the density of these cells did not substantially differ between the group of rats grafted with U87MG cells alone and the group grafted with U87MG cells plus MSCs as well as amongst rats of the same group.

## Discussion

MSCs are adult stem cells that have been isolated from several tissues and have recently gained great attention as a possible therapeutic tool for a number of diseases. The use of MSCs to treat cancer patients appears a real practical opportunity. Due to their unique ability to differentiate and to produce immunomodulatory as well as anti-apoptotic, anti-angiogenic, and pro-survival factors, MSCs gave promising results in a preclinical model of regenerative medicine [24]. However, the most relevant potentiality of MSCs in clinic stems from the peculiar feature of MSCs to home the stroma of various primary and metastatic tumors [25, 26]. This feature prompts using MSCs as vehicles to specifically deliver anti-tumor compounds directly to the tumor microenvironment, minimizing systemic toxicity. However, there is some evidence raising the issue of pro-survival effects of MSCs on tumor cells [27–29]. On the contrary, it has been demonstrated recently that induced pluripotent stem cell-derived MSCs possess lower potential to promote tumors than bone marrow MSCs [30]. We and others have previously demonstrated that MSCs of different origin can be loaded in vitro with anti-neoplastic drugs, like PTX and doxorubicin (DXR), without affecting their viability [16, 31–33]. The same does not hold true for other stem cells, such as hematopoietic stem cells, which display signs of cytotoxic damage after priming with DXR. We also showed that PTX-loaded MSCs are able to reduce tumor cell proliferation and endothelial cell viability in vitro and to reduce the growth of subcutaneous tumor xenografts [16, 31].

PTX is the best-selling chemotherapeutic compound in history [34] and is currently used to treat a wide variety of human cancers [35, 36]. PTX targets the β-tubulin subunit of microtubules in the cytoskeleton. Unlike other tubulin-targeting drugs such as colchicine that inhibit microtubule assembly, PTX stabilizes the polymer and protects it from disassembly [10, 12]. Chromosomes are thus unable to achieve a correct metaphase spindle configuration. This blocks progression of mitoses, and prolongs activation of the mitotic checkpoint triggering apoptosis [37] or reversion to the G phase of the cell cycle without cell division [38–40]. PTX-treated cells have defects in mitotic spindle assembly, chromosome segregation, and cell division. The ability of PTX to inhibit spindle function is generally attributed to its suppression of microtubule dynamics [41], but recent studies have demonstrated that suppression of dynamics occurs at concentrations lower than those needed to block mitosis [42]. At the higher therapeutic concentrations, PTX appears to suppress microtubule detachment from centrosomes, a process normally activated during mitosis [43]. The mechanism of PTX action and its in vivo cytotoxic effects therefore seem to depend mostly on its local concentration [14]. Moreover, PTX might also have a role in inhibiting angiogenesis and vascularization [44].

In this article we addressed two specific issues. Firstly, we wanted to prove that MSCs loaded with PTX retain their tropism towards the U87MG tumor cells. Several groups demonstrated that intracerebrally grafted MSCs migrate for considerable distances through the brain parenchyma approaching the site where GBM tumor cells have been implanted [17, 45]. Here, we wondered whether PTX loading may affect the tropism of MSCs towards GBM tumors. Secondly, we wanted to assess whether the brain xenografts containing PTX-loaded MSCs showed signs of PTX-induced cytotoxic damage by the U87MG tumor cells. Results answered both questions. In the U87MG brain xenografts, nearly all of the MSCs injected in the proximity of tumor cells migrated toward the tumor and populated it, and this behavior was not affected by PTX loading. The concentration of PTX achieved inside the tumor was high enough to induce a significant increase of abnormal spindle mitoses (mono/multispindles) and, as a consequence, of aberrant multinucleated cells.

PTX is commonly understood to act as a microtubule poison and to lead to mitotic arrest; however, this knowledge is largely based on in vitro studies. In a recent study, Zasadil et al. [14] measured the concentration of PTX in patients treated for breast cancer at therapeutic dosages. They found that at therapeutic concentrations of PTX the tumor cells do not undergo mitotic arrest but proceed through mitosis with abnormal spindles, resulting in chromosome missegregation, eventually leading cells to die. Our in vivo data are consistent with these observations. The use of MSCs for local PTX delivery does have a translational potentiality because we observed in vivo cytotoxic damage of the tumor cells similar to that obtained in patients after systemic administration. Importantly, our approach avoids the side effects of systemic PTX delivery without damaging the brain cell populations.

## Conclusion

The present work demonstrates that MSCs loaded with PTX maintain a tropism toward brain tumors, and that these cells release the drug causing a specific cytotoxic damage of the tumor cells. Our data support the value of MSCs for local anti-cancer therapy.

## Additional files

Additional file 1: Figure S1. Showing in vitro U87MG cell viability after treatment with CM from PTX-loaded MSCs. The kinetics of growth inhibition induced by serial 1:2 dilutions of CM from PTX-loaded MSCs on U87MG cells is compared with the growth inhibition of U87MG cells caused by PTX treatment at different concentrations (in the range of 1.56–200 ng/ml). The addition of CM induced a strong anti-proliferative effect in a dose-dependent fashion. Mean ± standard deviation are shown. (JPEG 293 kb) Additional file 2: Figure S2. Showing assessment of PTX-induced nuclear changes in the brain cell populations. Immunostaining either with GFAP A or with NeuN B showed that the nuclei of astrocytes (*arrows* in A) and of neurons (*arrows* in B) lying close to PTX-loaded MSCs do not exhibit those PTX-induced changes that are clearly seen in the nuclei of U87MG tumor cells (*arrowheads* in A and B). Scale bars = 25 μm. (JPEG 9615 kb) Additional file 3: Figure S3. Showing immunohistochemical characterization of microglial activation in brain tumor xenografts. Brain microglia was immunostained by Iba1 antibody. The grafted region of the brain shows a huge increase in microglial cell number with respect to the contralateral region of the brain (*right panels* versus *left panels*). No difference in microglial cell density is found between rats grafted with U87MG cells alone and rats grafted with U87MG cells plus MSCs (*upper panels* versus *bottom panels*). Scale bars = 50 μm. (JPEG 1312 kb)

## Abbreviations

ANOVA: Analysis of variance
BSA: Bovine serum albumin
CM: Conditioned medium
DAPI: 4′,6-Diamidino-2-phenylindole
DMEM: Dulbecco’s modified Eagle’s medium
DXR: doxorubicin
GBM: Glioblastoma multiforme
GFP: Green fluorescent protein
HBSS: Hanks balanced salt solution
IMDM: Iscove’s modified Dulbecco’s medium
MSC: Mesenchymal stem/stromal cell
NDS: Normal donkey serum
PB: Phosphate buffer
PBS: Phosphate-buffered saline
PTX: Paclitaxel
RT: Room temperature
SEM: Standard error of the mean
