# Supplementary figures and images for: Mesenchymal stromal cells loaded with paclitaxel induce cytotoxic damage in glioblastoma brain xenografts
Source: Stem Cell Res Ther. 2015 Oct 6;6:194. doi: 10.1186/s13287-015-0185-z (PMC4594910; doi:10.1186/s13287-015-0185-z)

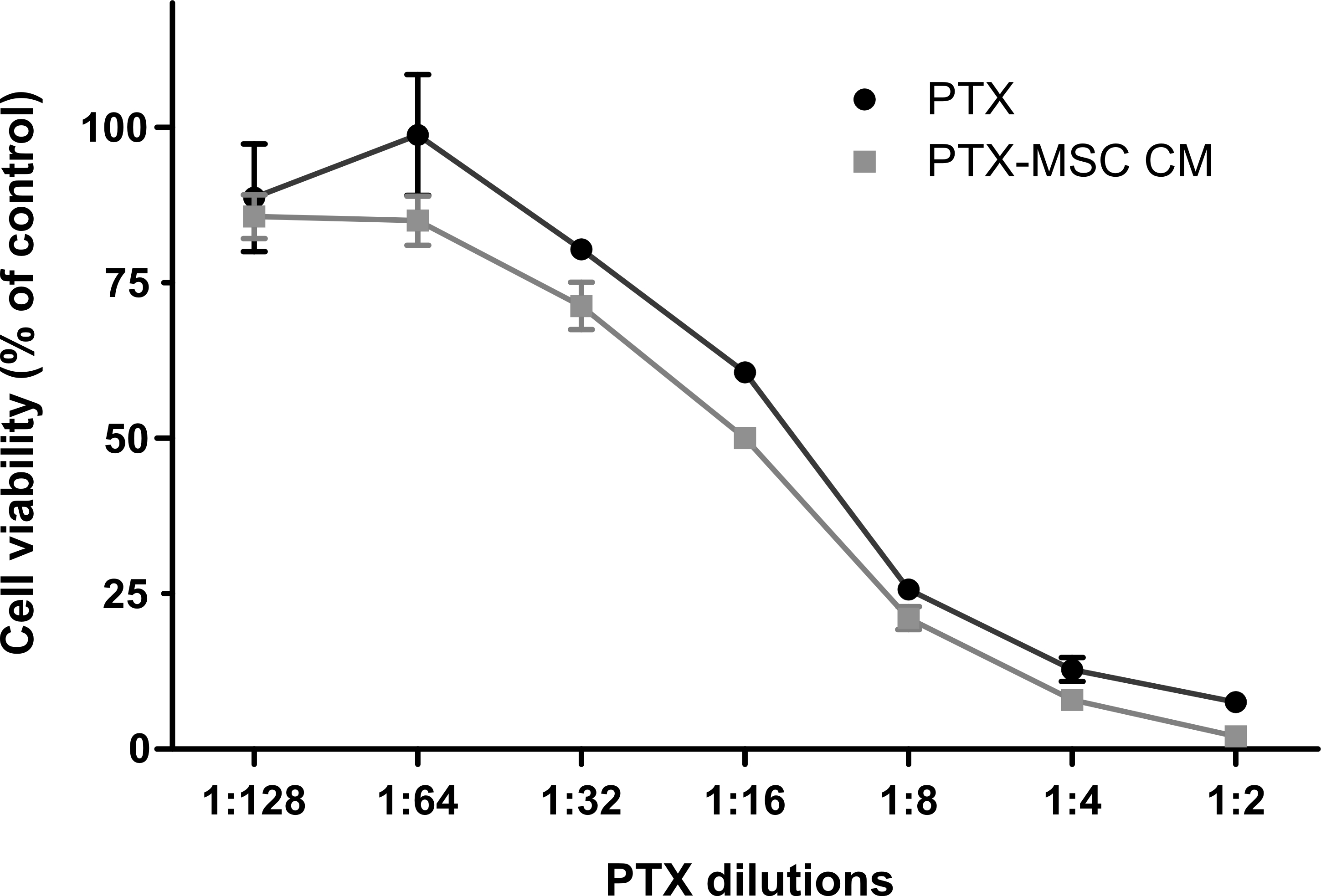

Supplement: Additional file 1: Figure S1. — Showing in vitro U87MG cell viability after treatment with CM from PTX-loaded MSCs. The kinetics of growth inhibition induced by serial 1:2 dilutions of CM from PTX-loaded MSCs on U87MG cells is compared with the growth inhibition of U87MG cells caused by PTX treatment at different concentrations (in the range of 1.56–200 ng/ml). The addition of CM induced a strong anti-proliferative effect in a dose-dependent fashion. Mean ± standard deviation are shown. (JPEG 293 kb) [file 13287_2015_185_MOESM1_ESM.jpg]

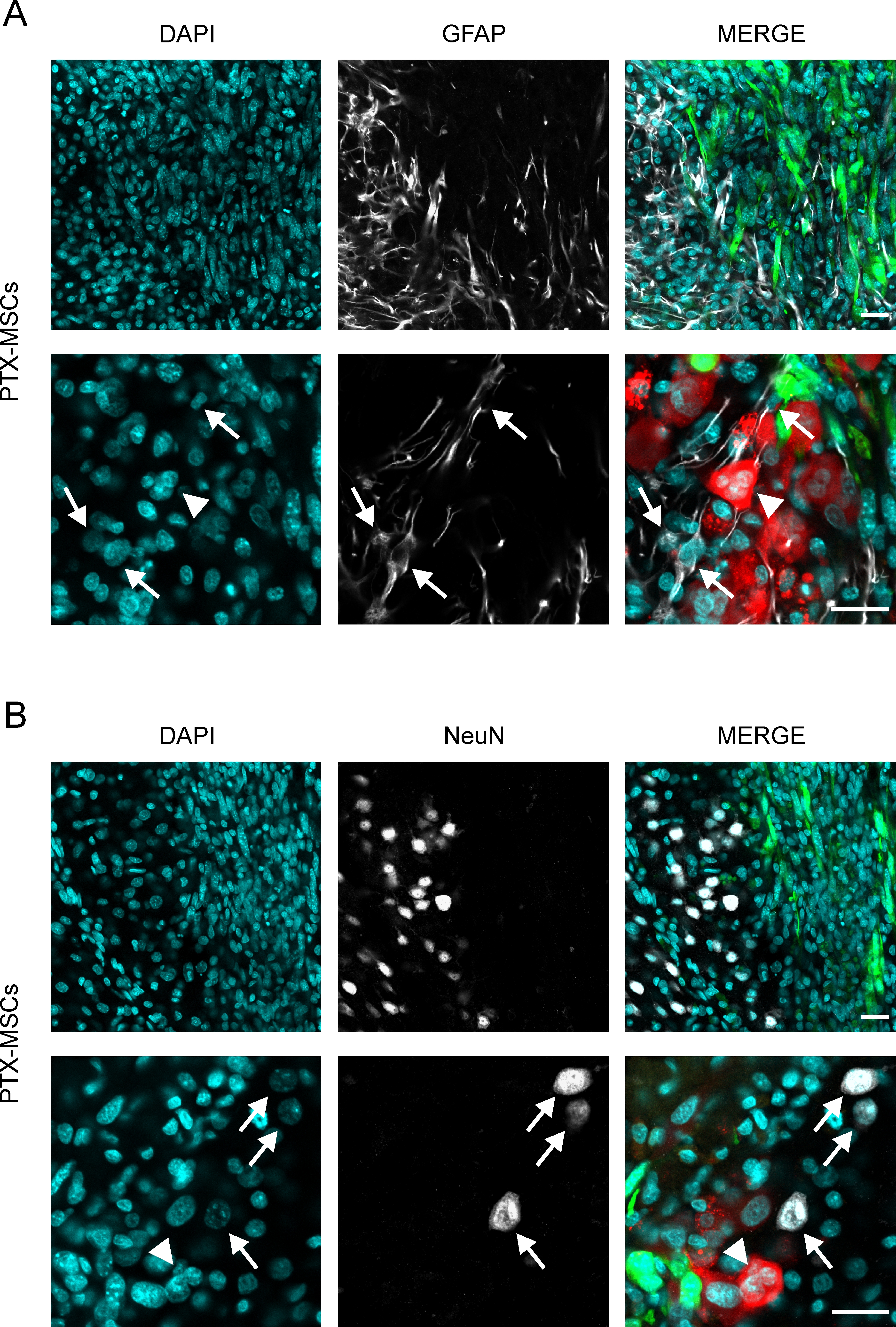

Supplement: Additional file 2: Figure S2. — Showing assessment of PTX-induced nuclear changes in the brain cell populations. Immunostaining either with GFAP A or with NeuN B showed that the nuclei of astrocytes (arrows in A) and of neurons (arrows in B) lying close to PTX-loaded MSCs do not exhibit those PTX-induced changes that are clearly seen in the nuclei of U87MG tumor cells (arrowheads in A and B). Scale bars = 25 μm. (JPEG 9615 kb) [file 13287_2015_185_MOESM2_ESM.jpg]

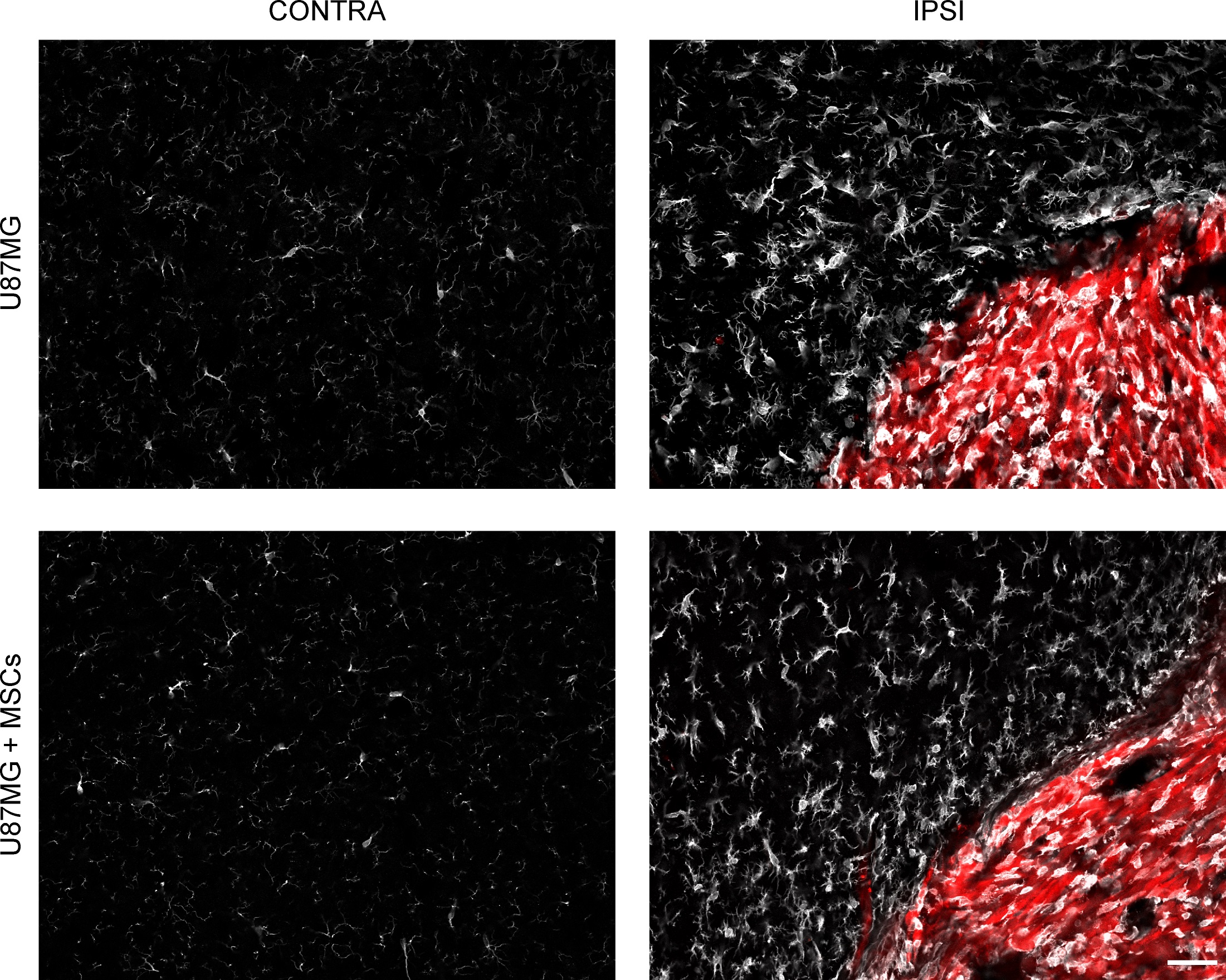

Supplement: Additional file 3: Figure S3. — Showing immunohistochemical characterization of microglial activation in brain tumor xenografts. Brain microglia was immunostained by Iba1 antibody. The grafted region of the brain shows a huge increase in microglial cell number with respect to the contralateral region of the brain (right panels versus left panels). No difference in microglial cell density is found between rats grafted with U87MG cells alone and rats grafted with U87MG cells plus MSCs (upper panels versus bottom panels). Scale bars = 50 μm. (JPEG 1312 kb) [file 13287_2015_185_MOESM3_ESM.jpg]
